# Comparison of sensitivity among dynamic balance measures during walking with different tasks

**DOI:** 10.1098/rsos.230883

**Published:** 2024-01-31

**Authors:** Shunsuke Yamagata, Takeshi Yamaguchi, Masahiro Shinya, Matija Milosevic, Kei Masani

**Affiliations:** ^1^ Institute of Sport Science, ASICS Corporation, Kobe, Japan; ^2^ Department of Finemechanics, Graduate School of Engineering, Tohoku University, 6-6-01 Aramaki-Aza-Aoba, Aoba-ku, Sendai, Miyagi 980-8579, Japan; ^3^ Graduate School of Biomedical Engineering, Tohoku University, Sendai, Japan; ^4^ Graduate School of Humanities and Social Sciences, Hiroshima University, Higashi-Hiroshima, Japan; ^5^ The Miami Project to Cure Paralysis, University of Miami, Miami, FL, USA; ^6^ Department of Neurological Surgery, University of Miami, Miami, FL, USA; ^7^ Department of Biomedical Engineering, University of Miami, Miami, FL, USA; ^8^ Institute of Biomaterials and Biomedical Engineering, University of Toronto, Toronto, Canada; ^9^ KITE Research Institute, University Health Network, Toronto, Canada

**Keywords:** dynamic balance, gait variability, Lyapunov exponent, margin of stability, whole-body angular momentum, desired centre of pressure

## Abstract

Although various measures have been proposed to evaluate dynamic balance during walking, it is currently unclear which measures are most sensitive to dynamic balance. We aimed to investigate which dynamic balance measure is most sensitive to detecting differences in dynamic balance during walking across various gait parameters, including short- and long-term Lyapunov exponents (*λ*_s_ and *λ*_l_), margin of stability (MOS), distance between the desired and measured centre of pressure (dCOP–mCOP) and whole-body angular momentum (WBAM). A total of 10 healthy young adults were asked to walk on a treadmill under three different conditions (normal walking, dual-task walking with a Stroop task as an unstable walking condition, and arm-restricted walking with arms restricted in front of the chest as another unstable walking condition) that were expected to have different dynamic balance properties. Overall, we found that *λ*_s_ of the centre of mass velocity, *λ*_s_ of the trunk velocity, *λ*_s_ of the hip joint angle, and the magnitude of the mediolateral dCOP–mCOP at heel contact can identify differences between tasks with a high sensitivity. Our findings provide new insights into the selection of sensitive dynamic balance measures during human walking.

## 1. Introduction

Human postural balance can be defined as the capability of maintaining an upright posture under the effect of perturbation due to external or internal factors, coming respectively from the environment or from the human body itself [1]. Human balance can be studied in static (e.g. during standing still) or dynamic (e.g. during walking) conditions. Our focus here will remain on dynamic balance and the associated dynamic balance measures. Various dynamic balance measures have been proposed to evaluate the risk of falling, and their effectiveness and validity have recently been compared in literature reviews [2,3]. Hamacher *et al*. [2] performed a comparative effect size (Cohen's *d*) analysis on the variability and dynamic stability of foot trajectories during level walking among young and elderly individuals based on 29 studies. The aforementioned systematic review identified balance measures that can discriminate between elderly and young individuals and between those more and less prone to falls among elderly individuals. Another study by Bruijn *et al*. [3] reviewed the nine currently available balance measures and assessed their validity, particularly in terms of construct validity, predictive validity in simple models, convergent validity in experimental studies and predictive validity in observational studies. We note that both studies are meta-analyses based on previous literature.

Various dynamic balance measures have been used to assess balance experimentally in healthy young adults, elderly individuals and individuals with gait disorders [4–12]. This research primarily focused on the dynamic balance under the effect of perturbation due to the internal factors, which are different from the external factors such as a slip on a slippery floor. Most of them have measured and compared multiple balance measures for different groups simultaneously within the same experimental conditions, but few have discussed which measure can be most sensitive to discriminate between stable and unstable gait. Therefore, it is still unclear which balance measure is most sensitive to identifying dynamic balance during walking, and the purpose of this study was to investigate which balance measures are more sensitive to discriminating stable and unstable gait.

In this paper, we selected five dynamic balance measures commonly used across many studies and one that we developed [13]. The dynamic balance measures included gait variability parameters [14–17] and the Lyapunov exponent [11,18–21], which are computed from temporal and kinematic data and derived from the dynamic system theory [3], and margin of stability (MOS) [22–27], distance between desired centre of pressure (dCOP) and measured centre of pressure (mCOP) (dCOP–mCOP) [13] and whole-body angular momentum (WBAM) [4,28,29], which are derived from biomechanics [3]. Among these, the traditional gait variability, Lyapunov exponent and MOS (including extrapolated centre of mass) were assessed as dynamic balance measures under unperturbed walking with good validity in the review paper by Bruijn *et al*. [3]. MOS, WBAM and dCOP-mCOP were able to discriminate the dynamic balance during walking between young and older adult groups in our previous study [6].

Gait variability, defined as stride-to-stride fluctuations while walking, has been closely associated with falls. Hausdorff *et al*. [14] reported that elderly individuals who had experienced a fall had a more unstable gait with larger variability in stride time and swing time than did those who had not fallen. The Lyapunov exponent is a measure that quantifies the degree of separation of two very close trajectories in state space and the local divergence of neighbouring trajectories in state space reconstructed from kinematic data [19], with large Lyapunov exponent values indicating a less stable system. A short-term Lyapunov exponent (*λ*_s_) is the initial phase of divergence, whereas a long-term Lyapunov exponent (*λ*_l_) is the terminal phase of divergence. Kang & Dingwell [19] indicated that elderly individuals had a larger *λ*_s_ of the trunk and pelvis than did younger ones, indicating that the trunk motion best represents the age-related differences in dynamic balance during walking. MOS is the distance between the extrapolated centre of mass of the whole-body (XCOM_body_) and the border of the base of support (BOS) [22]. XCOM_body_ is defined as the vector sum of the whole-body centre of mass (COM_body_) position and a proportion of its velocity. It is evaluated as stable if the projection point of XCOM_body_ is captured by the BOS and unstable if it is outside the BOS. Therefore, a larger MOS allows for greater system tolerance against perturbation in both anteroposterior (AP) and mediolateral (ML) directions [23]. dCOP is the virtual centre of pressure at which the sum of the moments acting on the COM_body_ during walking becomes zero when the actual centre of pressure (i.e. mCOP) coincides with the dCOP [13]. Therefore, the dCOP–mCOP is proportional to the magnitude of the moment acting on the COM_body_, and an increase in dCOP–mCOP results in an increase in the external moment around the COM_body_, potentially leading to the loss of postural balance. Yamaguchi *et al*. [13], who investigated dCOP–mCOP in a turning gait on a slippery floor, found that dCOP–mCOP in the lateral direction was a good predictor of falls. The WBAM is the sum of the angular momentum around the COM_body_ and the angular momentum of body segments [4,28,29]. In cases where the WBAM increases due to complex movements or where a decrease in the ability to regulate the WBAM exists, a higher level of balance control is required and the possibility of falls increases [28]. Vistamehr *et al*. [28] compared the WBAM between healthy adults and participants who had experienced a stroke and suffered from hemiplegia during straight walking and climbing steps. Notably, they found that individuals who had experienced a stroke had a larger frontal WBAM range.

By comparing these dynamic balance measures, we aimed to investigate which measures are most sensitive in detecting differences in dynamic balance between the following three walking conditions: normal walking, dual-task walking with a Stroop task as an unstable walking condition, and arm-restricted walking with arms restricted in front of the chest as another unstable walking condition. Arm swing during walking causes changes in WBAM [30] and walking speed [31]. It is also reported that the Lyapunov exponent is affected due to arm constraint [32]. Therefore, we hypothesized that arm-restricted walking is thought to affect biomechanical balance measures such as WBAM, MOS and dCOP-mCOP as well as balance measures related to local stability such as the Lyapunov exponent. On the other hand, it has been pointed out that dual-task walking during the Stroop test causes changes in trunk movement variability and Lyapunov exponent [33]. These findings suggest that traditional gait variability and Lyapunov exponent will be affected during dual-task walking, which is our second hypothesis. Since all the above findings were acquired in different experimental studies and have never been evaluated under same experimental conditions, we aimed to verify these hypotheses within the same controlled experimental paradigm. The abbreviations and symbols used throughout the paper are summarized in table 1.

**Table 1.** Nomenclature.

abbreviation/symbol	definition
AP	anteroposterior
AUC	area under curve
BOS	base of support
COM_body_	whole-body centre of mass, coordinates ($x_{{\mathrm{COM}}_{\mathrm{body}}},y_{{\mathrm{COM}}_{\mathrm{body}}},z_{{\mathrm{COM}}_{\mathrm{body}}}$)
CV	coefficient of variation
dCOP	desired centre of pressure, coordinates (*x*_dCOP_, *y*_dCOP_, 0)
D_DLS_	double-leg-stance duration
D_SLS_	single-leg-stance duration
*g*	acceleration of gravity = 9.8 ms^−1^
GRF	ground reaction force, coordinates (*F_x_*, *F_y_*, *F_z_*)
***H***	vector of whole-body angular momentum
*I*	moment of inertia of inverted pendulum consisting of whole-body centre of mass and measured centre of pressure, coordinates (*I_x_*, *I_y_*)
*I_i_*	moment of inertia of *i*th body segment
*l*	distance between the whole-body centre of mass and ankle marker
LHC	left heel contact
mCOP	measured centre of pressure
*m*_i_	mass of *i*th body segment
ML	mediolateral
ML Peak_HC_	peak value of mediolateral dCOP-mCOP at heel contact
MOS	margin of stability
MOS_HC_	margin of stability at heel contact
MOS_min_	minimum margin of stability during single-leg stance
*p*	probability
P_i_L_	*i*th peak dCOP–mCOP instance phase for left foot
P_i_R_	*i*th peak dCOP–mCOP instance phase for right foot
$r_{\mathrm{border}}$	position vector of the border of base of support
$r_{{\mathrm{COM}}_{\mathrm{body}}}$	position vector of whole-body centre of mass
$r_{{\mathrm{COM}}_{i}}$	position vector of centre of mass of *i*th body segment
RHC	right heel contact
$r_{\mathrm{MOS}}$	vector of margin of stability
RMS	root mean square
ROC	receiver operating characteristic
$r_{{\mathrm{XCOM}}_{\mathrm{body}}}$	position vector of extrapolated centre of mass of whole body
*S*(*t*)	state space
SL	stride length
SLS	single-leg stance
ST	stride time
SW	step width
*t*	time
*τ*	time delay
*v*	velocity, coordinates (*v*_x_, *v*_y_, *v*_z_)
$v_{{\mathrm{COM}}_{\mathrm{body}}}$	velocity vector of whole-body centre of mass
*v*COM_body_	velocity of whole-body centre of mass
*v*COM_foot_	velocity of centre of mass of foot segment
$v_{{\mathrm{COM}}_{i}}$	velocity vector of centre of mass of *i*th body segment
*v*COM_shank_	velocity of centre of mass of shank segment
*v*COM_thigh_	velocity of centre of mass of thigh segment
*v*COM_trunk_	velocity of centre of mass of trunk segment
WBAM	whole-body angular momentum
XCOM_body_	extrapolated centre of mass of the whole-body
*λ*_1_	long-term Lyapunov exponent
*λ*_s_	short-term Lyapunov exponent
$\ddot{\theta }$	angular acceleration around whole-body centre of mass, coordinates (${\ddot{\theta }}_{x},{\ddot{\theta }}_{y}$)
*θ_y_*	joint angle in the sagittal plane
*θ_y_*_ankle joint_	ankle joint angle in the sagittal plane
*θ_y_*_hip joint_	hip joint angle in the sagittal plane
*θ_y_*_knee joint_	knee joint angle in the sagittal plane
$\omega_{i}$	angular velocity vector of centre of mass of *i*th body segment

## 2. Methods

### 2.1. Participants

This study included 10 healthy young adult males whose age, height and body mass were 28.8 ± 6.0 years, 1.70 ± 0.06 m and 66.9 ± 7.2 kg (mean ± standard deviation), respectively. The participants had no history of neuromuscular, cognitive, musculoskeletal disorders or injuries that could affect their balance and walking ability. All participants were informed of the protocol and provided written informed consent prior to experiment. The protocol was approved by the ethics committee at the University of Tokyo.

### 2.2. Experimental procedure

The experimental set-up used in this study consists of a three-dimensional motion analysis system with seven infrared cameras (Qualisys Motion Capture Systems, Qualisys AB), an instrumented treadmill with two force plates embedded in each side (Instrumented treadmill FIT, Bertec Corporation) and a personal computer for data measurement. Infrared reflective markers were attached to 20 major joints throughout the participant's body (i.e. the ear, shoulder, elbow, wrist, anterior superior iliac spine (ASIS), trochanter, knee, ankle, fifth metatarsal and heel of each side), from which three-dimensional motion data were obtained. In addition, each force plate measured the ground reaction force (GRF) and mCOP independently for the left and right feet. The sampling frequencies for GRFs and three-dimensional motion data were 1 kHz and 200 Hz, respectively.

Participants were asked to walk on the treadmill for 10 min under three walking conditions: (i) normal walking, (ii) dual-task walking, and (iii) arm-restricted walking. The treadmill speed was set to 1.0 m s^−1^. During normal walking, participants were instructed to walk at their preferred stride length and cadence. During dual-task walking, participants walked while performing a cognitive task called the Stroop test [34], in which they answered aloud the colour of the letters displayed on a monitor set in front of them at eye level while walking. The letters were displayed on the monitor in intervals of 3 to 5 s. During arm-restricted walking, the participant walked with their arms crossed in front of their chest. Each condition was performed once at random order. To avoid fatigue effects, at least a 10 min break was provided between trials.

**Figure 1.**
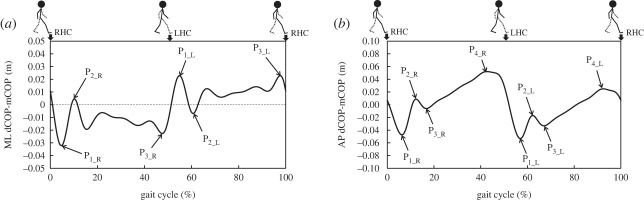
Temporal change in dCOP–mCOP during a gait cycle. RHC, right heel contact; LHC, left heel contact. *P_i_R_*, and *P_i_L_* represent *i*th peak dCOP-mCOP in stance phase for right and left feet. (*a*) ML direction. (*b*) AP direction.

### 2.3. Data analysis

The total number of steps during a 10 min trial in each task was about 1300 steps regardless of the participant. Stationary and non-missing data were analysed using 450 successive strides (900 steps) in the middle of each trial. Matlab (Mathworks, Natick, MA, USA) was used for subsequent analyses. Kinetic and kinematic data were low-pass filtered using a fourth-order Butterworth filter with zero lag and cut-off frequencies of 10 Hz. The position of the whole-body COM was estimated using a 12-segment model involving the motion data. Heel contact and toe-off were determined based on vertical GRF (greater than 50 N for heel contact and less than 50 N for toe-off, respectively).

#### 2.3.1. Dynamic balance measures

##### 2.3.1.1. Gait variability

Stride time (ST), single-leg-stance duration (*D*_SLS_), double-leg-stance duration (*D*_DLS_), stride length (SL) and step width (SW) were calculated using the heel contact and toe-off. ST was defined as the duration between two consecutive heel contacts of the same foot. *D*_SLS_ was defined as the duration between the toe-off and heel contact of the same foot. *D*_DLS_ was defined as the duration during double stance. SL was defined as the total length of two steps during a stride. SL was defined as the anteroposterior (AP) distance between the heel markers at heel contact. SW was defined as the ML distance between heel markers at heel contact. The mean value of the coefficient of variation (CV) of each variable was calculated.

##### 2.3.1.2. Lyapunov exponent

The three-dimensional time series of COM_body_ velocity (*v*COM_body_), COM_trunk_ velocity (*v*COM_trunk_), COM_thigh_ velocity (*v*COM_thigh_), COM_shank_ velocity (*v*COM_shank_) and COM_foot_ velocity (*v*COM_foot_) were used to calculate the Lyapunov exponent. The one-dimensional time series of the hip joint angle (*θ_y_*_hip joint_), knee joint angle (*θ_y_*_knee joint_) and ankle joint angle (*θ_y_*_ankle joint_) in the sagittal plane were also used. Data were resampled so that the number of data points per SL was 100 [35,36]. For each three-dimensional time series of each COM velocity data, a nine-dimensional state space *S*(*t*) was defined using time-delayed copies and embedding dimension,

2.1 $$\begin{array}{c} S(t)=[v_{x}(t)\;v_{y}(t)\;v_{z}(t)\;v_{x}(t+\tau )\;v_{y}(t+\tau )\;v_{z}(t+\tau )\;v_{x}(t+2\tau )\;v_{y}(t+2\tau )\;v_{z}(t+2\tau )], \end{array}$$

where *v_x_*, *v_y_* and *v_z_* are the velocities in the *x*, *y* and *z* axes, while *t* and τ are the time and time delay, respectively. For each one-dimensional time series of each lower limb joint angle, a six-dimensional state space was defined using time-delayed copies and embedding dimension,

2.2 $$\begin{array}{c} S(t)=[\theta_{y}(t)\;\theta_{y}(t+\tau )\;\theta_{y}(t+2\tau )\;\theta_{y}(t+3\tau )\;\theta_{y}(t+4\tau )\;\theta_{y}(t+5\tau )], \end{array}$$

where $\theta_{y}$ is the joint angle in sagittal plane. Time delays were calculated using the first minimum point of the average mutual information, and embedding dimensions were calculated using the false nearest neighbour method [37]. For each time series, the first minimum point of the average mutual information was calculated for each subject, gait condition and axis, and then averaged to obtain the time delay. The divergence curve was calculated using the algorithm proposed by Rosenstein *et al.* [38]. *λ_s_* was calculated as the slope between 0 and 0.5 strides, whereas *λ_l_* was calculated as the slope between 4 and 10 strides in the divergence curve [3,18].

##### 2.3.1.3. Margin of stability

The XCOM_body_ was defined as the vector sum of the COM_body_ position and a proportion of its velocity as follows [22]:

2.3 $$\begin{array}{c} r_{{\mathrm{XCOM}}_{\mathrm{body}}}=r_{{\mathrm{COM}}_{\mathrm{body}}}+\frac{v_{{\mathrm{COM}}_{\mathrm{body}}}}{\sqrt{g/l\;}}, \end{array}$$

where, $r_{{\mathrm{XCOM}}_{\mathrm{body}}}$ is the XCOM_body_ position, $r_{{\mathrm{COM}}_{\mathrm{body}}}$ is the COM_body_ position, $v_{{\mathrm{COM}}_{\mathrm{body}}}$ is the COM_body_ velocity, g is the gravity constant, and *l* is the distance between the COM_body_ position and ankle marker. The MOS was calculated as the distance between XCOM and the border of BOS as follows [22]:

2.4 $$\begin{array}{c} r_{\mathrm{MOS}}=r_{\mathrm{border}}-\;r_{{\mathrm{XCOM}}_{\mathrm{body}}}, \end{array}$$

where, $r_{\mathrm{border}}$ is the position of the border of BOS.

In this study, the MOS at the heel contact and the minimum MOS during single-leg stance were calculated [22–25,27]. ML MOS at heel contact (ML MOS_HC_) was defined as the distance in the ML direction between the XCOM_body_ and the heel marker position at heel contact. The minimum ML MOS during single-leg stance (ML MOS_min_) was defined as the minimum distance between the XCOM_body_ and the fifth metatarsal marker position during single-leg stance in the ML direction. AP MOS at heel contact (AP MOS) was defined as the AP distance between the XCOM_body_ and the heel marker position of the leading foot at heel contact. As to AP direction, only the MOS at heel contact was calculated given that the minimum AP MOS during single-leg stance agrees with that at heel contact. The mean value of magnitude normalized to the participant's body height was used for comparison.

##### 2.3.1.4. Distance between the desired and measured centre of pressure

The dCOP position (*x*_dCOP_, *y*_dCOP_) was calculated from the rotational motion equation of the inverted pendulum consisting of COM_body_ and mCOP. From the equation of rotational motion of the inverted pendulum in the frontal plane (*x–z* plane) and sagittal plane (*y–z* plane), the following equations were obtained [13]:

2.5 $$\begin{array}{c} I_{y}{\ddot{\theta }}_{y}=F_{z}(x_{{\mathrm{COM}}_{\mathrm{body}}}-x_{\mathrm{mCOP}})-F_{x}z_{{\mathrm{COM}}_{\mathrm{Body}}}\; \end{array}$$

and

2.6 $$\begin{array}{c} I_{x}{\ddot{\theta }}_{x}=F_{z}(y_{{\mathrm{COM}}_{\mathrm{body}}}-y_{\mathrm{mCOP}})-F_{y}z_{{\mathrm{COM}}_{\mathrm{Body}}}, \end{array}$$

where $I_{y}\;$and $I_{x}$ are the moment of inertia in the frontal plane and sagittal plane; ${\ddot{\theta }}_{y}$ and ${\ddot{\theta }}_{x}$ are the angular acceleration around COM_body_ in the frontal plane and sagittal plane; and $x_{{\mathrm{COM}}_{\mathrm{body}}},\;y_{{\mathrm{COM}}_{\mathrm{body}}}\;\mathrm{and}\;z_{{\mathrm{COM}}_{\mathrm{body}}}$ are the position of COM_body_ in the *x*, *y*, and *z* directions, respectively. For equations (2.5) and (2.6), when ${\ddot{\theta }}_{x},{\ddot{\theta }}_{y}=0$, the moment acting around COM_body_ becomes zero. Then, let $x_{\mathrm{mCOP}}=x_{\mathrm{dCOP}},y_{\mathrm{mCOP}}=y_{\mathrm{dCOP}}$, the dCOP position was calculated by the following equations:

2.7 $$\begin{array}{c} \;x_{\mathrm{dCOP}}=x_{{\mathrm{COM}}_{\mathrm{body}}}-\frac{F_{x}}{F_{z}}z_{{\mathrm{COM}}_{\mathrm{body}}}, \end{array}$$

and

2.8 $$\begin{array}{c} \;y_{\mathrm{dCOP}}=y_{{\mathrm{COM}}_{\mathrm{body}}}-\frac{F_{y}}{F_{z}}z_{{\mathrm{COM}}_{\mathrm{body}}} \end{array}.$$

The distance between dCOP and mCOP (i.e. dCOP–mCOP) throughout the whole gait cycle was calculated. The root mean square (RMS) during a single stride, range (max minus minimum) during the stride and RMS dCOP–mCOP during the single stance phase were calculated. We found that there were three and four unique peaks characterizing the dCOP–mCOP dynamics for ML and AP directions during single stance phase as shown in figure 1.

The mean value of each peak dCOP–mCOP normalized to the participant's body height was calculated and used for comparison.

##### 2.3.1.5. Whole-body angular momentum

WBAM was calculated using the following equation [28]:

2.9 $$\begin{array}{c} H=\;\overset{12}{\underset{i=1}{\sum }}[(r_{{\mathrm{COM}}_{i}}-r_{{\mathrm{COM}}_{\mathrm{body}}})\times m_{i}(v_{{\mathrm{COM}}_{i}}-v_{{\mathrm{COM}}_{\mathrm{body}}})+I_{i}\omega_{i}], \end{array}$$

where, $r_{{\mathrm{COM}}_{i}},\;v_{{\mathrm{COM}}_{i}},\;m_{i},\;I_{i}$ and $\omega_{i}$ are the COM position, COM velocity, mass, moment of inertia and angular velocity of *i*th body segment, respectively. The RMS of the WBAM during the single stride, range (maximum−minimum) of the WBAM during the single stride and the range of WBAM during the single stance phase were calculated. The mean value of each WBAM normalized by the product of the participant's body height, body weight and $\sqrt{g/l}$ were calculated for comparison.

### 2.4. Statistical analysis

We performed a paired *t*-test with Bonferroni correction to determine significant differences in balance measures between the three different walking tasks. The significant level was set at 0.05/3. The effect size for *t*-tests was also reported using Cohen's *d*, with values of 0.2–0.4, 0.4–0.8 and greater than 0.8 indicating small, moderate and large effects, respectively [39]. Receiver operating characteristic (ROC) analysis was also performed, after which the area under the curve (AUC) was calculated and used to investigate the discriminatory power of each balance measure between the normal walking task and the unstable walking tasks (i.e. dual-task and arm-restricted walking). An AUC between 0.5 and 0.7, between 0.7 and 0.9 and above 0.9 indicates low, medium and high discriminatory power, respectively [40].

Paired *t*-test and effect size analyses were performed using Microsoft Excel (Microsoft, Redmond, WA, USA). Effect size and ROC analyses were performed using Matlab version 8.3 (Mathworks, Natick, MA, USA).

## 3. Results

Table 2 summarizes the mean value of each balance measure for the three gait tasks and the results of the *t*-tests, effect sizes (Cohen's *d* values) and AUC values between normal, and dual-task or arm-restricted walking.

**Table 2.** Mean values of each balance measure for the three gait tasks and the results of the *t*-tests, effect sizes (Cohen's *d* values) and AUC values between normal and dual-task or arm-restricted walking.

measures			normal		dual task		arm restricted		normal versus dual task			normal versus arm restricted		
			mean	s.d.	mean	s.d.	mean	s.d.	*p*-value	Cohen's *d*	AUC	*p*-value	Cohen's *d*	AUC
Gait variability	ST	CV, %	1.86	0.38	1.93	0.38	2.37	0.54	0.970	0.184	0.530	*p* < 0.01	1.092	0.790
	D_SLS_	CV, %	3.16	0.72	3.39	0.68	4.09	0.82	0.175	0.328	0.625	*p* < 0.01	1.205	0.790
	D_DLS_	CV, %	5.66	0.59	5.90	1.25	6.13	1.11	1.801	0.246	0.450	0.590	0.529	0.560
	SL	CV, %	2.22	0.46	2.44	0.80	2.77	0.57	0.751	0.337	0.550	*p* < 0.01	1.062	0.790
	SW	CV, %	7.88	1.72	7.20	1.55	7.66	1.47	0.796	0.415	0.380	2.152	0.138	0.460
*λ_s_*	*v*COM_body_	magnitude, -	1.29	0.08	1.34	0.05	1.46	0.08	0.166	0.750	0.730	*p* < 0.01	2.125	0.910
	*v*COM_trunk_	magnitude, -	1.84	0.12	1.90	0.08	2.05	0.08	0.145	0.588	0.745	*p* < 0.001	2.059	0.870
	*v*COM_thigh_	magnitude, -	1.62	0.13	1.65	0.09	1.79	0.10	0.680	0.268	0.600	*p* < 0.05	1.466	0.840
	*v*COM_shank_	magnitude, -	1.67	0.11	1.70	0.09	1.80	0.11	0.818	0.299	0.550	*p* < 0.05	1.182	0.810
	*v*COM_foot_	magnitude, -	1.99	0.15	2.05	0.11	2.17	0.14	0.399	0.456	0.620	*p* < 0.05	1.241	0.790
	*θ_y_*_hip joint_	magnitude, -	1.08	0.07	1.11	0.09	1.23	0.06	0.042	0.372	0.620	*p* < 0.001	2.301	0.970
	*θ_y_*_Knee joint_	magnitude, -	1.29	0.05	1.30	0.07	1.37	0.06	1.528	0.164	0.560	*p* < 0.05	1.449	0.840
	*θ_y_*_Ankle joint_	magnitude, -	0.89	0.03	0.89	0.04	0.91	0.04	2.902	0.000	0.470	0.632	0.566	0.570
*λ_l_*	*v*COM_body_	magnitude, -	0.065	0.008	0.064	0.006	0.062	0.005	2.168	0.099	0.450	0.846	0.525	0.350
	*v*COM_trunk_	magnitude, -	0.066	0.008	0.065	0.007	0.063	0.005	1.554	0.186	0.450	0.949	0.465	0.390
	*v*COM_thigh_	magnitude, -	0.070	0.007	0.069	0.008	0.066	0.006	2.165	0.106	0.520	0.486	0.660	0.370
	*v*COM_shank_	magnitude, -	0.086	0.009	0.083	0.009	0.084	0.006	1.020	0.311	0.460	1.853	0.275	0.450
	*v*COM_foot_	magnitude, -	0.093	0.008	0.089	0.009	0.088	0.007	0.860	0.399	0.430	0.865	0.585	0.340
	*θ_y_*_hip joint_	magnitude, -	0.082	0.011	0.081	0.010	0.087	0.009	2.069	0.095	0.490	1.100	0.478	0.640
	*θ_y_*_knee joint_	magnitude, -	0.087	0.009	0.085	0.010	0.082	0.007	1.723	0.179	0.460	0.729	0.595	0.380
	*θ_y_*_ankle joint_	magnitude, -	0.063	0.004	0.061	0.009	0.062	0.007	1.074	0.302	0.530	2.186	0.210	0.440
MOS	ML MOS_HC_	magnitude, -	0.058	0.008	0.059	0.009	0.060	0.008	1.407	0.117	0.490	*p* < 0.05	0.263	0.580
	ML MOS_min_	magnitude, -	0.045	0.012	0.047	0.010	0.046	0.011	0.659	0.172	0.570	1.284	0.052	0.500
	AP MOS	magnitude, -	0.049	0.012	0.056	0.012	0.051	0.013	0.229	0.558	0.700	0.705	0.152	0.540
dCOP–mCOP	ML RMS	magnitude, -	0.010	0.002	0.010	0.002	0.011	0.001	1.065	0.250	0.620	*p* < 0.05	0.696	0.700
	ML SLS RMS	magnitude, -	0.009	0.002	0.010	0.002	0.011	0.001	0.554	0.300	0.600	*p* < 0.001	1.012	0.720
	ML Range	magnitude, -	0.039	0.007	0.040	0.004	0.045	0.004	1.617	0.193	0.610	*p* < 0.05	1.140	0.770
	ML Peak1	magnitude, -	0.017	0.004	0.017	0.002	0.018	0.003	2.733	0.032	0.560	1.385	0.198	0.580
	ML Peak2	magnitude, -	0.008	0.005	0.007	0.004	0.007	0.005	0.717	0.221	0.470	0.278	0.320	0.350
	ML Peak3 (ML PeakHC)	magnitude, -	0.016	0.003	0.017	0.003	0.021	0.002	0.287	0.467	0.620	*p* < 0.001	2.157	0.900
	AP RMS	magnitude, -	0.017	0.003	0.017	0.002	0.015	0.002	2.647	0.039	0.500	*p* < 0.001	0.784	0.320
	AP SLS RMS	magnitude, -	0.015	0.002	0.015	0.002	0.013	0.002	2.157	0.100	0.460	*p* < 0.01	0.950	0.280
	AP Range	magnitude, -	0.066	0.009	0.066	0.009	0.059	0.007	2.493	0.044	0.480	*p* < 0.001	0.905	0.240
	AP Peak1	magnitude, -	−0.031	0.006	−0.031	0.007	−0.027	0.006	2.070	0.000	0.510	*p* < 0.01	0.667	0.650
	AP Peak2	magnitude, -	0.000	0.006	0.000	0.006	0.004	0.006	2.704	0.017	0.470	*p* < 0.001	0.617	0.690
	AP Peak3	magnitude, -	−0.013	0.005	−0.012	0.005	−0.009	0.005	0.652	0.200	0.570	*p* < 0.001	0.800	0.760
	AP Peak4	magnitude, -	0.024	0.003	0.023	0.005	0.020	0.004	0.952	0.243	0.470	*p* < 0.01	0.990	0.290
WBAM	Frontal RMS	magnitude, -	0.005	0.001	0.005	0.001	0.005	0.001	1.793	0.117	0.550	2.352	0.150	0.520
	Frontal Range	magnitude, -	0.018	0.003	0.018	0.003	0.019	0.003	1.415	0.167	0.530	0.364	0.333	0.610
	Frontal Range_SLS_	magnitude, -	0.014	0.003	0.014	0.002	0.014	0.002	1.834	0.078	0.530	1.128	0.157	0.530
	Sagittal RMS	magnitude, -	0.005	0.001	0.005	0.001	0.004	0.001	0.830	0.200	0.470	*p* < 0.001	1.000	0.220
	Sagittal Range	magnitude, -	0.018	0.003	0.017	0.003	0.015	0.002	0.483	0.267	0.430	*p* < 0.001	1.059	0.230
	Sagittal Range_SLS_	magnitude, -	0.016	0.003	0.015	0.003	0.012	0.002	0.606	0.233	0.450	*p* < 0.001	1.216	0.220

The CV values of the ST, D_SLS_ and SL during arm-restricted walking were significantly larger than those during normal walking (*p*s < 0.05). The AUC values of these measures were found to discriminate between normal and arm-restricted walking with moderate discriminatory power (0.7 < AUC < 0.9). By contrast, no significant difference (*p* > 0.05) in the CV values of the gait parameters was observed between normal and dual-task walking and the AUC was < 0.7.

With regard to the magnitude of *λ_s_*, the mean *λ_s_* values for *v*COM_body_, *v*COM_trunk_, *v*COM_thigh_, *v*COM_shank_, *v*COM_foot_, *θ_y_*_hip joint_ and *θ_y_*_ankle joint_ were significantly larger during arm-restricted walking than during normal walking (*p*s < 0.05). The AUC values of these *λ_s_* values were found to discriminate between normal walking and arm-restricted walking with moderate (0.7 < AUC < 0.9) or high discriminatory power (AUC ≥ 0.9). λs value for *θ_y_*_hip joint_ was significantly larger during dual-task walking than during normal walking (*p* < 0.05). No significant differences in the magnitude of *λ_l_* were observed among walking tasks (*p* > 0.05).

With regard to the mean values of ML MOS, ML MOS_HC_ was significantly larger during arm-restricted walking than during normal walking (*p* < 0.05); however, ML MOS_min_ was not significantly affected by the gait tasks (*p* > 0.05). No significant difference in the mean value of AP MOS was observed among the gait task conditions (*p* > 0.05). The AUC values were less than or equal to 0.7 for three of MOS variables.

With regard to the mean values of ML dCOP–mCOP, ML RMS, ML RMS_SLS_, ML Range and ML Peak 3 appearing at heel contact (redefined as ML Peak_HC_) during arm-restricted walking were significantly larger than those during normal walking (*p* < 0.05). The AUC values of these measures were found to discriminate between normal and arm-restricted walking with moderate (0.7 < AUC < 0.9) or high sensitivity (AUC ≥ 0.9). No significant differences in the mean values of the ML dCOP–mCOP variables were observed between normal and dual-task walking (*p* > 0.05). With regard to the mean values of AP dCOP–mCOP, AP RMS, AP RMS_SLS_, AP Range and AP Peaks were significantly smaller during arm-restricted walking than during normal walking (*p* < 0.01); however, the AUC value was less than 0.5. The mean value of the AP Peak 2 was significantly larger during arm-restricted walking than during normal walking (*p* < 0.001). No significant differences in the magnitude and variability measures of the AP dCOP–mCOP were observed between normal and dual-task walking (*p* > 0.05).

No significant differences in the mean values of the frontal WBAM variables (Frontal RMS, Range and Range_SLS_) were noted among the gait task conditions (*p* > 0.05). With regard to the magnitude of sagittal WBAM variables, AP RMS, AP Range and AP Range_SLS_ were significantly smaller during arm-restricted walking than during normal walking (*p*_s_ < 0.001); however, the AUC values were less than 0.5. No significant differences in the mean values of the sagittal WBAM variables were observed between normal and dual-task walking (*p* > 0.05).

## 4. Discussion

### 4.1. Discriminatory power of dynamic balance measure

The results of the paired *t*-tests indicated a significant difference between normal and arm-restricted walking in terms of the CV value of ST, D_SLS_, SL, *λ_s_* of *v*COM_body_, *λ_s_* of *v*COM_trunk_, *λ_s_* of *v*COM_thigh_, *λ_s_* of *v*COM_shank_, *λ_s_* of *v*COM_foot_, *λ_s_* of *θ_y_*_hip joint_, *λ_s_* of *θ_y_*_knee joint_, ML RMS of dCOP–mCOP, ML RMS_SLS_ of dCOP–mCOP, ML Range of dCOP–mCOP, ML Peak_HC_ of dCOP–mCOP, and AP Peak3 of dCOP–mCOP (table 2). Moreover, these 15 measures were significantly larger during arm-restricted walking than during normal walking, suggesting that arm-restricted walking is more unstable than normal walking.

Among these 15 measures, 13, with the exception of the ML RMS of dCOP–mCOP, exhibited a large effect size (Cohen's *d* > 0.8), with *λ_s_* of *θ_y_*_hip joint_, *λ_s_* of *v*COM_body_, *λ_s_* of *v*COM_trunk_, and the ML Peak_HC_ of dCOP–mCOP exhibiting an especially larger effect size (Cohen's *d* > 1.8; table 2). With regard to the AUC, the 16 measures exhibited moderate discriminatory power (AUC > 0.7), with *λ_s_* of *θ_y_*_hip joint_, *λ_s_* of *v*COM_body_ and the ML Peak_HC_ of dCOP–mCOP exhibiting especially high discriminatory power (AUC > 0.90) (table 2). These results indicate that *λ_s_* of *θ_y_*_hip joint_, *λ_s_* of *v*COM_body_ and the ML Peak_HC_ of dCOP–mCOP can be used as highly sensitive measures in detecting differences in dynamic balance between normal and arm-restricted walking (unstable walking). These results support our first hypothesis, in which arm-restricted walking affects biomechanical balance measures and those related to local stability.

Paired *t*-tests results indicated that only *λ_s_* of *θ_y_*_hip joint_ was significantly larger during dual-task walking than during normal walking (table 2). No significant differences were observed in other balance measures between the normal and dual-task walking trials (*p* > 0.05). However, some of the balance measures showed a Cohen's *d* value of greater than 0.4 and an AUC of greater than 0.7, indicating that only a few significant differences were noted between dual-task and normal walking in the current study. This could be attributed to the possibility that the cognitive load of the Stroop test used in this study was insufficient for the participant population, and the memory required for processing the walking motion could be handled without interference. Thus, a larger cognitive load may have resulted in a significant difference in the balance measures. The results partially support our second hypothesis, in which dual-task walking during the Stroop test could affect the Lyapunov exponent but not the biomechanical balance measures.

The relatively high sensitivity of *λ_s_* of *θ_y_*_hip joint_, *λ_s_* of *v*COM_body_ and *λ_s_* of *v*COM_trunk_ may be explained by the fact that the trunk (50%) and upper leg (20%) occupy the majority of body mass that primarily affects dynamic balance. Maintaining trunk stability is suggested to be one of the most important aspects of dynamic balance [41,42]. Given that the hip joint connects the trunk and upper leg, its behaviours can also be critical in dynamic balance. Hamacher *et al*. [2] indicated that during level walking, the *λ*_s_ using trunk motion data can detect differences in local dynamic stability between older and younger populations with higher sensitivity, which supports our results.

Our results indicated that *λ*_s_ can detect gait instability with higher sensitivity than gait variability parameters and balance measures derived from biomechanics, such as MOS, WBAM and dCOP–mCOP. In fact, Bruijn *et al*. [3] confirmed the validity of gait variability measures and *λ_s_* for estimating gait stability during gait with a small perturbation without having large external mechanical perturbations. In the current study, small perturbations were continuously presented during walking with the unstable walking tasks. Gait variability measures (i.e. CV values of gait parameters) only quantify the average differences between strides, independent of the temporal order in which strides occur, indicating that such measures contain no information about how the locomotion system responds to perturbations either within or across strides. However, *λ*_s_ quantifies how fast gait patterns diverge after infinitesimal perturbation, which should exhibit a higher sensitivity compared with the gait variability measures used in the current study. This is also confirmed by Dingwell *et al*. [5], who showed that stride-to-stride variability and local dynamics stability quantify fundamentally different aspects of locomotor behaviour and that gait variability measures poorly predict local stability.

**Figure 2.**
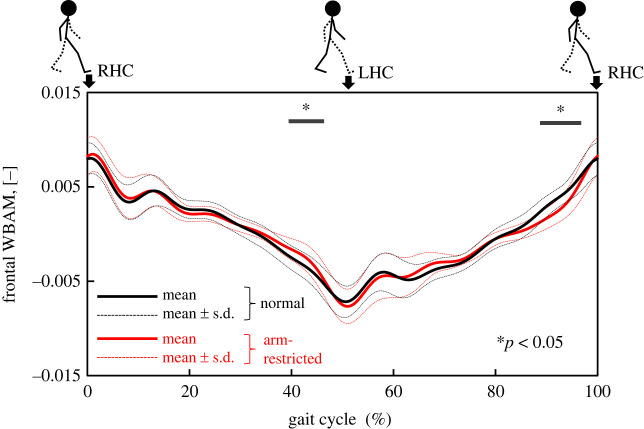
Comparison of the WBAM in frontal plane between normal walking and arm-restricted walking.

**Figure 3.**
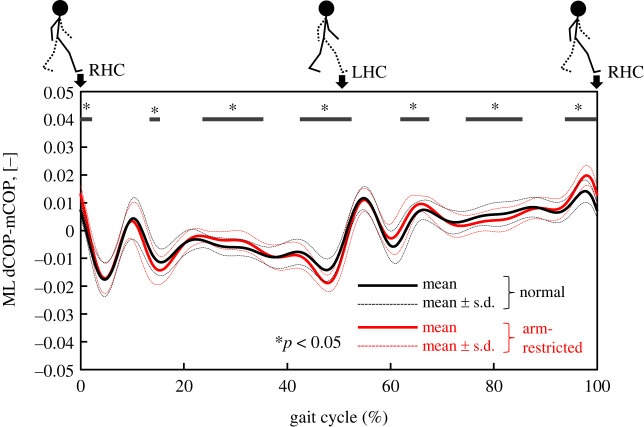
Comparison of the dCOP–mCOP in the ML direction between normal and arm-restricted walking.

Our results also suggests that the ML Peak_HC_ of dCOP–mCOP was able to identify differences in dynamic balance between normal and unstable walking. The derivative of WBAM is proportional to the dCOP–mCOP given that the derivative of WBAM is the moment around COM_body_, which is proportional to the dCOP–mCOP. Therefore, a rapid change in WBAM will appear as a peak in the dCOP–mCOP. As shown in figure 2, we performed multiple comparisons between normal and arm-restricted walking using the Benjamini–Hochberg method (BH method) [43] by 1% on the time series data of frontal WBAM normalized to the 0%–100% gait cycle. The figure shows a statistically significant difference (*p* < 0.05) at 39%–45% and 88%–95% (around heel contact). Similarly, as shown in figure 3, multiple comparisons were made using the BH method for the ML dCOP–mCOP between normal and arm-restricted walking. In the ML dCOP–mCOP, a statistically significant difference (*p* < 0.05) was observed at 42%–51% and 93%–100% (including the heel–ground contact). In the unstable phase before heel contact, the WBAM changed constantly in normal walking, whereas the absolute value of the WBAM during arm-restricted walking increased rapidly. In the phase just before heel contact, it would be difficult to control the WBAM during arm-restricted walking, and the moment around the COM_body_ increased.

MOS is theoretically regulated by step length and SW in the AP and ML directions, respectively [22,23]. We confirmed that the mean values of SL and SW did not significantly differ among gait tasks (*p* > 0.05), which could be attributed to the miniscule perturbations in each unstable walking condition. This could be the reason why MOS could not identify a difference in the dynamic balance between the normal and unstable walking tasks performed herein.

### 4.2. Study limitations

Some limitations of the current study should be considered. First, our study was conducted on a treadmill; hence, it is unclear whether our results can be applied to overground walking. Second, because the arm-restricted walking condition can be a highly unstable gait configuration, the results obtained in this study may not be applied to unstable gait in general. Another limitation was that the participants were all young adult males. Therefore, whether our results could be applied to dynamic balance alterations associated with disability or ageing needs to be further investigated. Although we investigated effect size, the small sample size (n = 10) could limit our results. As such, further studies with a larger sample size are needed. While our reflective marker placement is sufficient for extracting the parameters analysed in the current study, future work should consider if the observations demonstrated herein would work with standard marker placement (e.g. Helen Hayes marker set).

## 5. Conclusion

This study has been an attempt to compare dynamic balance measures, such as gait variability measures, Lyapunov exponents, margin of stability, desired centre of pressure and whole-body angular momentum, through identical gait trials with different gait tasks (normal, dual-task and arm-restricted walking). The findings of the current study indicate that *λ*_s_ of *v*COM_body_, *λ*_s_ of *v*COM_trunk_, *λ*_s_ of *θ_y_*_hip joint_ and the magnitude of the ML Peak_HC_ of dCOP–mCOP are dynamic balance measures with high sensitivity for detecting difference in dynamic balance between normal walking and unstable walking tasks. Our findings provide new insights into the selection of more sensitive dynamic balance measures and relationships among dynamics balance measures.

## Data Availability

This article has no additional data.
